# Perceptions and Challenges of Engineering and Science Transfer Students From Community College to University in a Chinese Educational Context

**DOI:** 10.3389/fpsyg.2021.797888

**Published:** 2022-01-27

**Authors:** Yui-yip Lau, Yuk Ming Tang, Nicole S. N. Yiu, Ceci Sze Wing Ho, Wilson Yeung Yuk Kwok, Kin Cheung

**Affiliations:** ^1^Division of Business and Hospitality Management, College of Professional and Continuing Education, The Hong Kong Polytechnic University, Hong Kong, Hong Kong SAR, China; ^2^Department of Industrial and Systems Engineering, The Hong Kong Polytechnic University, Hong Kong, Hong Kong SAR, China; ^3^Department of Civil and Environmental Engineering, The Hong Kong Polytechnic University, Hong Kong, Hong Kong SAR, China; ^4^School of Nursing, The Hong Kong Polytechnic University, Hong Kong, Hong Kong SAR, China

**Keywords:** Hong Kong higher education, sub-degree, transfer students, transition process, transition adjustment

## Abstract

In Hong Kong, transfer students encounter different challenges unfolding in their transition from community college to university study. However, limited research has been conducted to explore their discipline-specific challenges. To address this gap, in this study three engineering and science faculties were selected from which to collect data through 35 in-depth interviews with transfer students, followed by a thorough thematic analysis. With the concept of in-betweenness, three main themes were identified: (1) “shifted the focus of study” (three sub-themes: (i) academic excellence in community college; (ii) future career in the university; and (iii) university life); (2) “encountered challenges in the transition” (four sub-themes: (i) non-matching program articulation; (ii) heavy study workload and its associated consequences; and (iii) non-specific administration arrangement and support from university); and (3) “students’ voices to enhance learning experiences” (four sub-themes: (i) modify the study duration; (ii) improve program articulation; (iii) improve social adjustment; and (iv) overseas exchange). The results of this study indicate the challenges faced by transfer students in their transition from community college to university and have implications for universities to design and implement appropriate strategies to prepare for the future.

## Introduction

Community colleges, technical colleges, junior colleges, private universities, and public universities are categorized commonly as higher education institutions (Belfield and Bailey, 2011). However, their missions, visions, histories, organizational structures, and expectations are usually heterogeneous (Kelly-Kleese, 2004). Worldwide, starting from the early 1980s, there has been a marked trend of changing from elite to mass higher education (Chen, 2008). As a consequence, the higher education sector has expanded dramatically. The huge expansion of community colleges has led to a remarkable increase in engagement in higher education. Of particular note is the growing inclusion of students whose chances to continue education beyond high school have been limited in the past due to various factors causing their academic disadvantage (Goldrick-Rab, 2010). In principle, community colleges introduce “open enrollment,” “open-door policies,” or “open admission” policies for students who have graduated from high school. The aim of these policies is to democratize the chances for every student, irrespective of prior disadvantages or advantages (i.e., a non-selective approach). In other words, community colleges need to make concerted efforts to assist disadvantaged students to attain the next academic level (Goldrick-Rab, 2010), which is university education. According to Starobin et al. (2016), the role of the community college is to ensure that students are well-equipped and ready academically to transition to university. Transfer students who are higher diploma or associate degree graduates with credit transfers are usually admitted to a 2-year pathway to finish their undergraduate programs in university (Lau et al., 2018). A smooth student transition depends on integrative efforts by the administrations of both the community college and the university, such as to improve the transfer agreements, and to organize the transfer partnerships and senior peer mentoring (Starobin et al., 2016). In fact, community colleges and universities should work together to facilitate students’ steady integration into new relationships, roles, and daily routines during the process (Elliott and Lakin, 2020). University personnel should be aware of transfer students’ needs and how they can adapt better to the new environment (Starobin et al., 2016).

However, to date, most of the research about transfer students has been conducted in Western contexts and there have only been a few studies focusing on Asian contexts, particularly in the Chinese contexts. Most existing studies have targeted transfer students in general, but those from different disciplines might have different needs. The transition experience can start at the time of moving from community college to university, with an understanding of the students’ expectations of the coming challenges and comprehensive planning ahead (Elliott and Lakin, 2020). However, to date, there is little research about these perceptions and challenges. This study addressed the existing gaps, particularly for transfer students in engineering and science fields in a Chinese context. The objectives of the study were to: (1) explore transfer students’ expectations for their ongoing community college transition process; (2) identify the students’ challenges throughout the transition process, and (3) identify the possible solutions to enhance their learning experiences.

## Literature Review

Australia, Canada, Hong Kong, India, Malaysia, Philippines, United Kingdom, and United States (US) provide various study pathways from community college to university (Farnsworth and Cissell, 2006; Wang, 2012; Lee, 2014; Archambault, 2015; Ong and Cheong, 2019; Bowker, 2021). However, the relevant studies are mainly from the United States and have not, therefore, addressed the specific needs of transfer students in Asian contexts, for instance, Chinese students. In the Chinese culture, students believe education has a high value and serves as a means to upward mobility (Lee, 2019). In the education system, “elitism” has been a deep-rooted concept held by students, parents, teachers, and academic institutions for over a century. Pubic examinations are used to distinguish between superior and inferior students. Therefore, Chinese students generally desire to continue their studies directly from high school to university in order to achieve a better place in society (Lee, 2019). This means that those who fail to achieve the scores required for university admission can see themselves as “losers” due to the pressure from different parties like parents, peers, and society in general (Ching et al., 2020). Although community colleges provide an alternative route to university, the students who take this path still perceive themselves as losers compared to mainstream students who enter university directly from high school (Wong, 2019, 2020; Ching et al., 2020). In addition, there is an expectation in Chinese society that students should complete their education before joining the workforce (Gu, 2006). Consequently, many who cannot achieve university entry will enroll instead in community college studies, with the aim of articulating to university 2 years later, via the new and innovative “2 + 2” pathway. Thus, unlike their counterparts in the US, where more transfer students tend to enter the workforce first and then return to study when they are older, in Chinese societies the ages of transfer and mainstream students are similar (Lee, 2014; Cheung et al., 2020b). The demographics of the transfer students in the United States can be very diverse. They might be married with children, working concurrently with their studies or having some years of working experience, which means more diversity in their ages as well. Some researchers have suggested that the age difference between non-transfer (traditional) and transfer students might be a factor contributing to the challenges the latter have in establishing social networks and peer relationships during their transition process (Fematt et al., 2021). However, with age difference being less of an issue in Asian contexts, the transfer students’ social challenges could be different. These potential differences have not been studied well to date.

Some previous research studies (Cheung et al., 2020a,b; Ching et al., 2020; Elliott and Lakin, 2020) have addressed transfer students’ transitional challenges or uncertainties, such as sense of belonging, motivation to study, academic workload stress, transfer shock [i.e., drop in Grade Point Average (GPA)], management of transition stress, mental health, and access to resources or learning support services. These challenges might be explained by the concept of in-betweenness (Bhabha, 1996; Dai, 2020; Dai and Hardy, 2021). Unlike mainstream students, transfer students encounter changes of an in-between space across the study environment from community college to university. These changes not only involve physical campus environment, but also teaching and learning pedagogy and even social and supporting culture. Frelin and Grannas (2010) addressed that teachers encounter the challenges of building informal interaction with their transfer students in the context of in-between spaces. In addition, changes that occur in the in-between space can affect students’ personal development (Bhabha, 1996; Dai, 2020; Dai and Hardy, 2021). Vilsmaier and Lang (2015) and Olin-Scheller et al. (2021) also pointed out that students look for feedback and support for sustainability learning in the in-between spaces. The complicated transition processes of moving from community college to university space may create turbulent pathways for transfer students. They can feel confronted by adjusting to their new environmental, academic, and psychological contexts (Umbach et al., 2019). Hence, these students may have poor completion rates or lack commitment to becoming involved in university life (Goldrick-Rab, 2010). Even though there is some information about these undesirable outcomes, and the potential challenges that can occur during the transition process (Cheung et al., 2020a,b; Ching et al., 2020; Elliott and Lakin, 2020), there has been little exploration of their expectations and views of the transition experience. Moreover, studies of transfer students have either involved students from multiple disciplines (Cheung et al., 2020a,b,c) or focused on just one discipline, such as nursing (Ching et al., 2020), or hospitality and tourism (Chan et al., 2021). There is a paucity of studies describing the transitional experiences of transfer students from engineering and science. One study examined the success factors (Jackson and Laanan, 2015), and another evaluated the role of advising officers in supporting or hindering the transfer pathways (Packard and Jeffers, 2013). In order to provide discipline-related support for this group of students, this study explored the perceptions and challenges experienced by transfer students from engineering and science in the Asian context of Hong Kong.

## Method

### Research Context

This study was conducted in Hong Kong, where Chinese ethnicity occupies 92% of the population (GovHK, 2021). In general, mainstream and transfer students are the two main types of students enrolling in undergraduate programs. Mainstream students (non-transfer students) are those who are admitted to the 4-year bachelor’s programs directly from high school. However, in Hong Kong, only about one-third of high school students could directly admit to the eight government-funded universities (Concourse, 2021; Hong Kong Examination and Assessment Authority (HKEAA), 2021). As such, there are large numbers of students who fail to gain direct entry into university from high school, and thus select community college programs as an alternative route to university education. These are referred to as transfer students. It is a remarkable general practice in Hong Kong that the government-funded universities reserve a predetermined number of places for community college associate degree and higher diploma holders to complete their bachelor degrees within 2 years, following the “2 + 2” full-time study pathway. Although the quota for such approved places has increased by over 101.05% over the past 10 years (Information Portal for Accredited Post-Secondary Programmes (iPASS), 2021), there is still keen competition for a large number of community college graduates to take this alternative pathway. To articulate vertically to undergraduate studies, they not only require competitive GPAs, but also need to perform well in admission interviews. In normal cases, community college students are usually admitted to the university as 3rd-year students in the 4-year bachelor degree program structure. This means their study duration is the same as the mainstream students’ (Ching et al., 2021). In addition, students who are studying in the university in this study are required to complete a certain credit of general education requirement subjects to fulfill the graduation requirement. These subjects include English and Chinese language, service learning, general education, and work-integrated education.

### Participants

This research was conducted in a public-funded university in Hong Kong. This University generates the largest number of vertical transfer students of all Hong Kong’s public-funded universities. Purposive sampling was used to recruit participants from engineering and science departments from three faculties (hereafter referred to by the pseudonyms of Faculties A, B, and C). The selection criteria included (i) students who had graduated from a 2-year community college program and studied in a 2-year bachelor program; and (ii) those who had been studying in the bachelor’s degree program for at least one semester. As expected, the respondents had already become part of and familiar with the learning environment. We recruited the appropriate participants via in-class and email promotion to boost the participation rate. Focus group interviews were conducted depending on the participants’ availability. As suggested by Gall et al. (2003), semi-structured interviews were considered to be more suitable than using structured interview guides or informal, chatty interviews. Semi-structured interviews can begin with predetermined questions and then provide the interviewers with chances to raise probing questions as a follow-up, or to change the questions based on the focus of the responses (Turner, 2010). In addition, the adoption of semi-structured interviews helped the participants to share their perceptions and experiences freely.

### Data Collection

Twelve sessions of focus group interviews and two individual interviews were arranged with 35 transfer students (18 female and 17 male). The arrangement of the number of students in one interview depended on students’ availability to fit their busy schedules. In order to facilitate the interview process and create a cohesive atmosphere, the focus groups were organized based on their disciplines or faculties. Depending on the participants’ availability, these group sizes ranged from two to four participants. Each interview was conducted in a quiet room within the university and lasted for 60–120 min. Each respondent was only invited to participate in one interview. The interview arrangement is summarized in Table 1.

**TABLE 1:** Information about interviews.

Faculty	Number of interviewees	Number of sessions	Date of interview
Faculty A	8	4	May to October 2018
Faculty B	21	8	March to November 2018
Faculty C	6	2	March to September 2018

Institutional ethical approval was obtained (i.e., HSEARS20170808003) before commencing the interviews. The interviewers were experienced and well-trained to conduct qualitative studies in higher education. However, they did not know the participants, which could perhaps have influenced the reliability of the research findings. Prior to the interview, each participant was given an information statement explaining the research background, purpose and format, the interview duration, and complaint procedures. The participants were assured that their personal particulars would be kept highly confidential, and they could withdraw at any point in time, without penalty or loss of benefits (Sewerin and Holmberg, 2017). After reading the statement, the participants were invited to sign a consent form.

We employed an interview guide with questions designed to examine the transfer students’ learning experiences (Table 2). This was based on a review of considerable literature relating to our research objectives (e.g., Pascarella, 1985; Hagedorn et al., 2006; Laanan et al., 2010). The interview guide was discussed with panels of experts and researchers to identify appropriate content and design in order to ensure the validity of the content, avoid double-barreled questions, and minimize fuzzy wording (Lau et al., 2021a). The interviewers first asked a general, broad question and followed this by raising a series of probing questions.

**TABLE 2:** Interview guide to explore the transition experiences of transfer students.

General broad question	(1) Can you tell me about your experience studying in the university till now?
Probing questions	(1) What is your perception of your transition experience at the university? (2) Have you encountered any needs and challenges throughout the whole transition? What is your perception about them? (3) What is your perception of the differences and similarities between yourself and other transfer students/non-transfer students?

The interviewers encouraged communication and facilitated discussion among the group members in Cantonese, the participants’ first language. All interviews were digitally recorded. In order to improve the accuracy, at least one research assistant attended each focus group as an observer and jotted down notes relevant to the interviewees’ communications. After each interview, the research assistant and the interviewers conducted debriefing sessions or review meetings to cross-check the interview notes and discuss the key points. The data analysis occurred concurrently with the collection. We proceeded with interviewing new participants until a point of data saturation was reached (Morse, 1995).

### Data Analysis

In general, the key philosophical methods of educational research can be divided into four main paradigms, namely postmodernism, positivism, interpretivism, and critical theory. A paradigm presents a key group of philosophical perspectives of the nature of the world. Also, a paradigm produces a philosophical plan of the key social and educational research patterns (Pring, 2004). In practice, interpretive research and qualitative research are associated closely with each other. In terms of interpretive investigators, a comprehensive understanding of the fundamentals of the impacts of social life and human behavior in a natural context can be acquired from qualitative research (Habermas, 1972). This study used qualitative research to gain a richer and deeper understanding of the meanings people attribute to actions and incidents and explore the complicated scenarios encountered by industry practitioners and researchers.

The audio-recordings of the interviews were transcribed verbatim in Chinese and then imported into NVivo Pro 12 for data management and analysis. Each participant was randomly assigned a code, using an anonymous form for identification (i.e., 14A means interviewee A for the 14th interview). We used a thematic analysis to identify the common themes, topics, ideas, and patterns of the collected qualitative interview findings. As expected, thematic analysis can “reflect our view of qualitative research as creative, reflexive and subjective, with researcher subjectivity understood as a resource, rather than a potential threat to knowledge production” (Braun and Clarke, 2019, p. 591). Braun and Clarke (2006) defined thematic analysis as “identifying, analyzing, and reporting patterns (themes) within data. A thematic analysis of the transcribed responses to the open-ended questions enabled a thorough exploration of engineering and science transfer students’ perceptions of the university learning environment and the challenges they experienced (Castleberry and Nolen, 2018), since thematic analysis provides a method that needs transparency, theoretical knowingness, and reflexivity (Braun and Clarke, 2019).

According to Yin (2009), the thematic analysis framework consists of five key steps: compiling, disassembling, reassembling, interpreting, and concluding. First, we took the time to compile the interview transcript data. It was important to allow plenty of time for this process as the researchers needed to comprehend the data by reading and rereading them word by word and line by line. Second, we disassembled the data for coding purposes. Austin and Sutton (2014) described coding as “the process by which raw data are gradually converted into usable data through the identification of themes, concepts, or ideas that have some connection with each other.” In the coding process for this study, the codes were given by tags, labels or names so as to categorize the information and recognize similarities and differences in the data. We asked experienced researchers, research assistants, and interviewers to review the coding in order to assess it, remove duplications and integrate identical codes. This enabled us to increase objectivity and credibility. In brief, this step transformed the analysis from inductive to deductive (Sutton and Austin, 2015). The deductive analysis is a method of employing theory from the start of research projects as sensitizing concepts and hypotheses to test (Creswell and Plano, 2011). Third, we performed a reassembly process to map or categorize the coding. In doing so, we created themes according to a pattern of codes and possibly separated them into subthemes. Fourth, we interpreted the data, which was the starting point for the conclusions. Basically, fairness, credibility, relatedness, completeness, and accuracy are the five critical success factors for exceptional qualitative interpretation. After the third step, we concentrated on addressing the thematic patterns of the data associated with the key research questions (Yin, 2009). We were able to identify the differences and similarities between the research context and background to decide the connection and suitability of the findings (Collingridge and Gantt, 2008).

## Results

Three themes and eleven sub-themes were identified (Table 3). The first, second, and third themes respectively addressed RQ1: Explore transfer students’ expectations of their ongoing community college transition process, RQ2: Identify the transfer students’ challenges throughout the transition process, and RQ3: Identify the possible solutions to enhance transfer students’ learning experiences.

**TABLE 3:** Summary of themes, subthemes, and codes.

Themes	Sub-themes
1. Shifted the focus of study	1.1. Academic excellence in community college (Codes: Learning attitude and concern on the academic result)
	1.2. Future career in the university (Codes: Career development support; career’s information accessibility; eligibility of internship; professional qualification; updated industry knowledge; focus on career aspect; and practical knowledge and skills)
	1.3. University life (Codes: Peer relationship; learning environment; and learning experience)
2. Encountered challenges in the transition	2.1. Non-matching program articulation 2.1.1 Transfer from the same discipline (Code: Misaligned course contents) 2.1.2 Transfer from a different discipline (Codes: Same workloads for transfer and non-transfer students; different study paths and levels; and different study backgrounds) 2.1.3 Study duplication (Code: Overlapped study area)
	2.2 Heavy study workload and its associated consequence 2.2.1 Limited spare time (Codes: Continuous assessment; examinations; and higher stress resistance) 2.2.2 Limited social/university life (Codes: Heavy study workload; credit transfer information; and credit transfer transparency) 2.2.3 Limited engagement (Codes: Lack of interaction opportunities; transfer and non-transfer students study separately; and perception of subject choices)
	2.3. Non-specific administration arrangement and support from the university (Codes: Unfair registration system; learning support; learning materials accessibility; facilities accessibility; and online system)
3. Students’ voices to enhance the learning experience	3.1. Modify the study duration (Code: Risk of deferment)
	3.2. Improve program articulation (Codes: Restriction on subject selection and Irrelevant study area)
	3.3. Improve social adjustment (Codes: Cohesion with a peer; cohesion with academic staff; and cultural conflict)
	3.4. Overseas exchange (Codes: Exchange opportunities; exchange support; and financial support)

### Shifted Focus of Study

Transfer students tend to take programs in community colleges as a “stepping stone” or a “second chance” for further university study (Lau et al., 2018). According to the concept of in-betweenness (Bhabha, 1996; Dai, 2020; Dai and Hardy, 2021), transfer students build on the academic excellence from the community college to develop themselves further in university.

#### Academic Excellence in Community College

Generally, the students who had studied in community colleges had to study very hard to achieve excellent academic results to compete for university entry due to the high academic requirements for the limited transfer places. They placed much emphasis on academic performance, and tended to ignore the development of personal interests. Jenkins and Fink (2016) further explained that the transfer process exhibits a competitive and severe screening system that retains only the strongest sub-degree holders.

*When I was studying the sub-degree, I had to obtain as high a GPA as possible*… *to increase the chance to transfer into university*. (34A, Yr1, Faculty A).

*I did not have much college life (during the sub-degree), I spent most of my time on studying, because the GPA requirement is high for the articulation from sub-degree to bachelor’s degree*. (30B, Yr1, Faculty B).

*When I was a sub-degree student, I focused on the academic aspect and was not keen to participate in the extra-curriculum and social activities, because getting the university place was my target.* (40A, Yr1, Faculty A).

#### Future Career in the University

After transferring to the university, most of the transfer students expected that university study could improve their employability, based on the belief that university life provides plenty of opportunities and support to broaden their horizons and enrich their experiences for career development, such as internship programs. In order to keep an excellent quality of education, the university may need to revamp programs to add value with regard to professionalization in higher education (Lau and Ng, 2015). To this end, human capital fosters and sustains higher education and produces new talent in the industry (Becker, 1993).

*My expectation is to enhance my professional knowledge in this university program, and to know more about the career path in this discipline, to prepare myself to take the right direction after graduation*. (30A, Yr1, Faculty B).

*Our department provides an internship for working as a technician or designer in an industry. We can experience our future working environment. Also, at some time, the internship company might employ us after we have graduated.* (34B, Yr1, Faculty A).

*I think attending the lessons and the internship provided by the university can enable us to try to experience the future working environment and enhance our career goals after graduation.* (29B, Yr1, Faculty C).

#### University Life

In addition, the transfer students had expected to explore university life by making more friends and participating in extra-curricular activities.

*I want to meet more friends here, I also want to enjoy university life and not only focus on studying.* (34B, Yr1, Faculty A).

*As a transfer student, I want to have more time to join extra-curriculum activities* (32B, Yr2, Faculty B).

The idea of social presence is important for students to establish social relationships among their classmates during university life (Garrison et al., 2000). To address this need, teachers can create learning communities of students on various social media platforms or tools (e.g., WhatsApp, Facebook, Twitter, and LinkedIn) to encourage them to have useful learning experiences and keep friendships (Lau et al., 2021a,b).

### Encountered Challenges in the Transition

The concept of in-betweenness (Bhabha, 1996; Dai, 2020; Dai and Hardy, 2021) can be applied to the transition experiences of transfer students. The degree of program articulation between community college and university plays a significant role in the learning experiences of transfer students in the university.

#### Non-matching Program Articulation

##### Students Transferred From the Same Discipline

Even within the same discipline, transfer students expressed different views and encountered different challenges. For example, in relation to academic issues, some found the subject content in the university program to be more difficult. They needed to spend time self-studying to recap the knowledge, due to gaps in the content of their sub-degree and university programs.

*Some subject content does not align with our previous program. I have to spend more time studying the basic knowledge, and it is difficult. (44A, Yr1, Faculty B).*

*I think there is a gap in content between sub-degree and university. I need to spend more time to take up the new content, but I am still not able to understand the new knowledge fully*. (06C, Yr1, Faculty C).

However, some students in Faculty A had a different view about their subject content. They had learned advanced knowledge in some subjects in their sub-degrees, but they had to take the same subjects at the introductory level again when they transferred to the university. Some of them felt that this was a problem caused by the inadequate articulation alignment.

*I have studied some subjects which were more difficult than the introductory level, but I had to study these introductory levels again after transferring to the university. I think it is meaningless. I thought these subject credits could be transferred, but they were rejected (by the university).* (20A, Yr2, Faculty A).

*I think what I have learned (after transfer to the university) is just introductory level, I think some advanced knowledge should be taught, but our teachers are adjusting it to an easier level.* (43B, Yr2, Faculty A).

##### Students Transferred From Different Disciplines

Some transfer students were transferred from their sub-degrees in disciplines that were different from those in which they enrolled at university. They found it challenging to study a new discipline and indicated that the learning content was not aligned to their previous sub-degrees. They needed to invest more in acquiring the knowledge of the new discipline.

*We have to take two subjects about which we did not have any relevant knowledge in the first semester (after transferring to university). Non-transfer students who study in a 4-year program can take one subject each semester, so they can have more time to understand the knowledge thoroughly.* (34A, Yr1, Faculty A).

*Our teachers assume all of us are science students but, indeed, many of us are non-science students. Some teachers might assume we have some relevant knowledge and thus they do not teach the basic knowledge again*. (32B, Yr2, Faculty B).

*I think some subjects in the first year are difficult. The university might assume all of the transfer students have basic knowledge of this discipline, indeed, and we do not have any relevant background as we did not study the related sub-degree.* (29B, Yr1, Faculty C).

Thus, it is understandable that some transfer students experienced heavier workloads because of the irrelevance of their academic backgrounds before transferring to the university.

*We, transfer students, have a heavy workload, because some of us did not study the relevant discipline before transferring to the university and were even without any related background for the current program. The university assumes all transfer students have basic knowledge in this discipline. We might need to learn about the new technology which other non-transfer students have been learning about before, but we are required to submit assignments quickly, so we feel it is so difficult to study here.* (43A, Yr2, Faculty A).

##### Study Duplication

Another issue is that some transfer students who had transferred from the same disciplines in which they had studied in their sub-degrees indicated that some subject content was a repetition of what they had learned in their sub-degrees. They felt disappointed to be studying the same content and expected to learn new knowledge after transferring to the university.

*I thought what I learned in the sub-degree was easier and expected that what I learned in university would be more difficult. However, I found that the contents of the sub-degree and university are the same*…. *It seems like I did not learn anything in these 2 years.* (20B, Yr2, Faculty A).

*In fact, we have already studied this content during our sub-degree. And now, we need to study it again. I think it is not a good articulation.* (31C, Yr2, Faculty B).

*I had learned about this subject and similar content in my sub-degree. I do not understand why I was unable to transfer the credit for this subject.* (29A, Yr1, Faculty C).

To a certain extent, students who had transferred from the same or different disciplines commonly indicated that non-matching program articulation adversely impacted their learning progress and demotivated their learning. As suggested by Ching et al. (2021), curriculum coordinators and administrators from universities and community colleges may form working groups and round table discussions leading toward constructive solutions (e.g., creating articulation agreements for enhancing credit transfer) that reduce transfers students’ study loads. Moreover, insufficient chances to obtain new knowledge during a class or the duplication of content were described as students’ main excuses for not attending classes. To this end, educators may need to revamp their programs to improve students’ learning motivation (Takase et al., 2019).

#### Heavy Workload and Its Associated Consequence

##### Limited Spare Time

The transfer students commonly perceived more study stress, heavier workloads, and greater coursework demands after transferring to the university. As well as the articulation issues mentioned in section “Non-matching Program Articulation,” which increased their study workloads, they also indicated that the university curriculum study schedule was more complex than in the sub-degree, with more examinations, assignments, and extra credit requirements. Ching et al. (2020) and Chan et al. (2021) also found that transfer students encounter heavy study workloads but shorter study periods. The majority of subjects are exam-oriented, which induced more study stress in the transfer students. The transfer students also need to comply with graduation requirements like job placements and service-learning. In doing so, they are being squeezed by packed schedules.

*I have more examinations after transferring to the university.* (34B, Yr1, Faculty A).

*I perceived higher study stress after transferring to university because I have a lot of things to do. Sometimes I feel that I cannot complete it by the deadline.* (42E, Yr1, Faculty B).

*I think the study schedule of the university curriculum is tighter than before. Since I am a transfer student, I have to fulfill approximately 64 credits plus other extra subjects*. (06A, Yr1, Faculty C).

##### Limited Social and University Life

The transfer students wanted to have more time to enjoy their university lives and join extra-curriculum activities, but they also understood that they had limited time to spend on social life because of the restricted timeframe of the “2 + 2” pathway and the heavy study workload. Wang and Wharton (2010) also addressed the issue that transfer students engage less in social activities like student organizations and involvement in student support services. In addition, some of the respondents implied that getting involved in social activities might have negative impacts on their academic performances.

*We have too many tasks (academic assessments) to do, we do not have much time free to join the university activities. Basically, it is hard to arrange our time to join the activities*. (43A, Yr2, Faculty A).

*We are already year three and four, we have no time to enjoy ourselves*. (43B, Yr2, Faculty A).

*We have many academic assignments to do, I have set a lower expectation for myself like a GPA not lower than 2.0 (out of 4), that is fine for me, so that I have been able to join some university activities and I feel happy to join, but the time is really limited. However, sometimes I feel guilty about getting a lower GPA because my peers are spending most of their time in study*. (42A, Yr1, Faculty B).

*The non-transfer students have more time to enjoy their university lives, like joining the societies. For us, we have only 2 years, in fact, we do not have much time to enjoy ourselves.* (29C, Yr1, Faculty C).

Also, they perceived difficulties associated with joining the university activities, particularly the overseas exchange, because they found that the exchange program was not designed for transfer students. There were several hidden restrictions for them. For instance, they had only a few choices for exchange places and would potentially delay their graduations due to the insufficient overseas exchange credit transfer. In accordance with Cheung et al. (2020c), excessive study loads meant that transfer students did not have a chance to engage in overseas exchanges.

*When I received the study plan and the timetable in admission, I knew that I would not have a chance to go on an overseas exchange. For transfer students, due to the heavy workload, final year project, and credit issues, we do not want to defer our graduation so that we know that we have no chance to go on an overseas exchange. Exchange is only for non-transfer students.* (34B, Yr1, Faculty A).

*Some of my classmates applied (for the overseas exchange), but they were not successful because we have a limited application period and choices for the overseas exchange. Only a few transfer students want to exchange in the final year, because normally we are not able to transfer the credit we earn during the exchange, and that will delay our graduation*… *we think that the overseas exchange is not designed for transfer students*. (30A, Yr1, Faculty B).

*Transfer students have less chance (to go on exchange), because there are quotas of only one or two places in each school and that might not be suitable for us. For example, I am studying aviation, but the exchange program is related to IT engineering, which is not directly related to my discipline. That means, if you go to exchange, you have to defer your graduation.* (29C, Yr1, Faculty C).

##### Limited Engagement

The students explained that they had studied sub-degree and undergraduate programs in two different settings. Poor social adjustment and engagement in the new (university) environment are common challenges for transfer students. Some researchers (e.g., Mehr and Daltry, 2016) described transfer students’ encounters with the “campus culture shock” of an unknown and new learning context. In a new learning environment, friendship is crucial to provide adolescents with camaraderie, enrichment, social skills training, and emotional support (Hiatt et al., 2015). In the interviews, the transfer students said that they had faced obstacles to making new friends with non-transfer students, as both groups of students have few interactions with each other. They also described difficulties with fitting into the new study environment where many social relationships and friendships had been already established.

*I think the bonding with the classmates in my sub-degree is stronger; we were always together, having lessons, and joining activities. But after transferring to the university, I always stay alone, or just with one or two friends. It is difficult to meet new classmates here. Especially, I think it is difficult to make friends in the environment where the classmates have been studying together for 2 years, and they have already built their friendships. It is difficult for me to get into their circles and groups*. (34A, Yr1, Faculty A).

*Transfer and non-transfer students tend to form groups by themselves during group work. In fact, they already have their own social circles and common topics. In turn, non-transfer students prefer to work among themselves*. (44A, Yr1, Faculty B).

*I have limited opportunities to interact with non-transfer students. The non-transfer students prefer to be with other non-transfer students as they have similar study paths and backgrounds. I find it difficult to join in their group as they have already formed their cohesion with one another. In addition, I also have limited time.* (29A, Yr1, Faculty C). Some transfer students said that they feel more comfortable being friends with their transfer student peers because they have similar backgrounds and hence find it easier to connect with each other.

*I think some non-transfer students are not friendly; they present like they are different from us and look down on us. Thus, we are not keen to be friends with them. But, of course, not all of the non-transfer students are the same*. (20B, Yr2, Faculty A).

*To be honest, I think there is a gap between non-transfer and transfer students.* (30B, Yr1, Faculty B).

*I think it is easier to connect and communicate with the transfer students; it might be because we have similar backgrounds and experiences.* (06C, Yr1, Faculty C).

#### Non-specific Administration Arrangements and Support From the University

The interviewees observed that the arrangements in the university are not designed for transfer students. Most of the transfer students reflected their dissatisfaction with the subject registration system. They indicated that they were the last group of students to register in the system. They had limited choices of subjects since those in which they were interested were already filled by the non-transfer students who have the higher priority in the subject registration system. They felt that they have more restrictions than the non-transfer students. Townsend and Wilson (2006) argued that transfer students were difficult in adjusting to the stringent academic standards of universities. The common problems have been arisen from registering for suitable subjects and transferring credits obtained from their particular community colleges to university studies.

*For transfer students, our study plan is tight. For example, we take the service-learning subject (a graduation requirement for all undergraduate students) in the last year, when our workload is heavy plus the final year project. However, it is impossible for us to register for the serving learning subject earlier because it is very competitive (in the registration system).* (40A, Yr1, Faculty A).

*Transfer students normally have tight study schedules, and this has already reduced our choices of the subjects we want to take. Also, we are not given any priority to take the subjects in the registration system, and finally we only have a few choices and usually we have to take subjects we are not interested in*. (44A, Yr1, Faculty B).

*We have only 2 years to complete the general education requirement subjects, which often clash with our discipline subjects’ timetables*… *the registration period is earlier for non-transfer students than for us in the subject registration system.* (29C, Yr1, Faculty C).

The transfer students reported that the university does not provide adequate support to them. They also pointed out that they have different, unique needs, for instance, transfer transition and credit transfer. To address this, universities may need to develop thorough support systems to facilitate transfer students to finish their undergraduate programs smoothly and successfully (Chan et al., 2021).

*I think for the students transferring from the sub-degree in the same university, the adjustment is smooth, because they are used to studying in a similar format. But those who transfer from different community colleges need more support because they are not familiar with the new environment. They do not know where they can get support, such as some welfare or the location of facilities.* (40A, Yr1, Faculty A).

*There is not enough support for helping our adjustment to university. The current university resources are provided to the 4-year program students or JUPAS-admitted students. Unlike non-transfer students, we had already completed the 2-year sub-degree program; since we already have 2 years’ experience of tertiary education, the university expects we are able to adjust quickly to the new campus life, so the support for us is less.* (45, Yr2, Faculty B).

*I think we need support regarding credit transfer. For example, I had taken some subjects in community college which were similar to some subjects in this university, but I was unable to transfer credit*. (06A, Yr1, Faculty C).

### Students’ Voices About How to Enhance the Learning Experience

#### Modify the Study Duration

Owing to their heavy workloads, the transfer students suggested that the university extend their articulated program to one more semester or a year, and provide a wider choice of one-semester service learning subjects. They agreed that extending the study period could enhance their learning outcomes and shorten the subject duration from two semesters to one, which could relieve their tight study schedules. This is consistent with the report by Chan et al. (2021) that transfer students desired to extend their normal program duration because of the tightly packed program structure. It also needs to be remembered that students perceive higher education to be a risky investment. Deferment of studies may induce students to bear higher education costs and lose job opportunities (Rey, 2012).

*I suggest adding one more semester to our program, which would benefit our study.* (42A, Yr1, Faculty B).

*I think we should have one more year, i.e., to have 3 years to complete our program. If we had one more year to study, we could enjoy our studies and not just fulfill the academic requirements.* (06B, Yr1, Faculty C).

*I think the university could provide more one-semester-long service-learning subjects. There are too many two-semester subjects*. (43A, YR, Faculty A).

#### Improved Program Articulation

The transfer students mentioned they had found some issues with the program articulation, such as duplicated learning content and different levels of learning content between sub-degree and university programs. Xu et al. (2016, p. 37) described that “the transfer pathway is “insufficiently structured and supported,” owing to the lack of agreements on credit transfer and articulation between community colleges and universities.” Thus, they suggested the university should revise program content in order to improve the articulation between sub-degree and university. Also, they suggested the university should consider students’ different academic backgrounds and organize some foundation subjects to enhance their basic knowledge, to improve their articulation.

*I suggest the university should review the program structure - like the content of the subject - and consider whether the content will overlap with the sub-degree or not.* (20B, Yr2, Faculty A).

*I suggest (to the university) to revise the content. Some of the content of the bachelor’s program is the same as that in the sub-degree program.* (31C, Yr2, Faculty B).

*I would suggest to organize some subjects that teach basic knowledge, like one or two subjects; this would be helpful for us (transfer students to have better articulation).* (06B, Yr1, Faculty C).

#### Improve Social Adjustment

The transfer students suggested that the university or individual faculties could take initiatives to organize a series of social activities to encourage their engagement with the new study environment and their connections with their non-transfer counterparts. They thought it would be significant to have a supportive learning context and teaching approaches to enhance the linkage with other students. Importantly, social activities can help students to have high personal expectations to achieve high academic performance via the process of knowledge building (Orooji and Taghiyareh, 2018; Ching et al., 2020).

*If we really do not have time to join the extra-curriculum activities, I think departments can organize some activities for us, like only within our own departments or even collaborating with others, to provide a platform for all students to meet. For example, like for Halloween, we can have a party or gathering or something like that*. (34B, Yr1, Faculty A).

*The faculty or university could arrange more activities to break the ice between transfer and non-transfer students, such as camping. I think that ice-breaking would be fantastic and contribute to my personal growth* (32C, Yr2, Faculty B).

*We do not have chances to get into the non-transfer students’ circles. Our teachers could randomly assign and mix up non-transfer and transfer students to form groups for projects. Otherwise, we normally form a group with other transfer students*. (29A, Yr1, Faculty C).

#### Overseas Exchange Opportunities

As mentioned above, most transfer students agreed that the overseas exchange program is not designed or suitable for them. In order to minimize the possible risks before deferring their studies and graduating on schedule, transfer students are forced to give up overseas exchange opportunities. In other words, the programs failed in meeting their initial expectations (Ching et al., 2021). Chew and Croy (2011) reinforced that overseas exchange should be considered as incentives for tertiary students to enroll in programs and pursue studies, The students in this study suggested that the university could modify program settings to increase their opportunities to join the exchange.

*I think the university could provide more summer exchange programs for transfer students, like in a shorter duration, which would be suitable for transfer students as they would find it easier to handle their time, and increase their chances of joining the exchange.* (34A, Yr1, Faculty A).

*I think the only way to help transfer students to join the exchange would be to allow them to do the serving learning subject overseas*. (45A, Yr2, Faculty B).

*I think the major concerns for transfer students to overseas exchange are insufficient credit transfer and delayed graduation. Thus, improving the credit transfer for the exchange program could encourage us to join the exchange.* (29C, Yr1, Faculty C).

## Discussion and Conclusion

Our study is one of the only such studies to explore the perceptions and challenges of community college transfer students in engineering and science disciplines during their university study in a Chinese education context. Unlike mainstream students, transfer students have new “in-between” characteristics which give rise to some unique challenges for them in the new learning environment, when they transfer from community college to university studies. Our qualitative study found three themes and eleven subthemes relating to the students’ shift in the focus of their study from community college to university, their expectations of university study, challenges experienced, and possible solutions to enhance their transition.

### Shifted Focus of Study and Expectations After Transferring to the University

The transfer students experienced a critical time during their community college studies, when they had to work hard to achieve outstanding academic performances in order to gain university places. The high academic requirements of university articulation are consistent across Asian contexts, such as Malaysia (Ong and Cheong, 2019) and Vietnam (Le, 2013). In Hong Kong, in particular, university places are limited and highly competitive (Kember, 2010). Students who are unable to obtain these places directly from high school are considered as academic failures, causing them high levels of stress (Wong, 2019, 2020). Thus, most community college students invest a lot of effort in pursuit of university places (Kember, 2010; Monaghan and Attewell, 2015). The transfer students interviewed in our study described their expectations of transferring to the university, which included expecting to enjoy university life and enhance their employability. They looked forward to enjoying university life after having spent most of their time studying in community college. This lack of social life for community college students has also been reported in the United States previously. The difference is that, rather than spending time studying, some United States counterparts have family and work commitments (Berger and Malaney, 2003). On the other hand, similar to our Chinese students, some Latino community college students in the United States do not prioritize participating in extra-curricular activities. Since their primary goal is to complete community college study, they tend to participate in activities which are beneficial to their academic goals (Zell, 2010). Likewise, some other studies have reported community college students treating their community college studies as a stepping stone to transfer to bachelor’s programs, therefore not wanting to engage in social activities or even not finding it necessary to spend time on-campus events (Borglum and Kubala, 2000). Therefore, it is not surprising that transfer students aspire to participate in social activities after transferring to the university. In fact, Berger and Malaney (2003) provided support that transfer students were more likely to spend time on social activities after transferring to the university.

Currently, employers are unwilling to arrange remedial skill development or on-the-job training and, hence, transfer students must rely on further education to improve their employability (Chhinzer and Russo, 2018). As suggested by Betts et al. (2009) and Chhinzer and Russo (2018), universities need to allocate available resources to support students’ development of the knowledge, skills, and attitudes required for their future careers. Internship programs need to offer the best methods for students to accumulate valuable working experience and obtain entry-level professional skills before joining the job market (Gault et al., 2000). Students transferring to university have been shown to gain additional chances to “acquire skills and qualifications” to improve their employability (Cheung et al., 2020a). Thus, the transfer students interviewed in this study expected that the university would provide strong career development, support, and various overseas exchange opportunities that would enhance their employability.

### Challenges Encountered in the Transition and Possible Solutions Suggested by Students

The level of alignment between community college and bachelor’s program is still an unsolved issue (National Academies of Sciences, Engineering, and Medicine, 2016). This different level of articulation between community college and university programs creates a different level of in-between transfer students. In our study, the transfer students found that there was a disparity in the content of the two programs (community college and university). In fact, community colleges have been regarded as having lower academic standards (Peng, 1978). For example, Townsend (1995) reported that most transfer students found the academic standards in university to be higher and the subject content harder than in community college. Additionally, Eggleston and Laanan (2001) found that, for at least half of the students transferring from technical community college programs that technical-based backgrounds might weaken their adjustment to the elite academic-based university programs. However, surprisingly, some of our transfer students in Faculty A reflected the opposite view about their subject content. They indicated that some of their community college content was more difficult than that in university. Although there have been only a few relevant studies investigating the alignment of learning content in different disciplines, some researchers have found that changes in students’ academic performances after they have transferred to university might vary in different disciplines (Cejda, 1997; Cejda et al., 1998). These findings implied that the learning experiences of transfer students from different disciplines might be variable. Apart from disparities in the content of subjects, there was also evidence in our current study of content duplicated in community college and bachelor’s programs. This was a novel finding demonstrating issues regarding the level of alignment across institutions. All this, together, suggests that university and faculty administrations should revise the curriculum content and review the alignment between the two programs, in order to improve the transfer students’ learning outcomes and the overall quality of the curriculum. Furthermore, some of the transfer students in our study had switched to different majors after transferring to the university and reflected their difficulties in studying new knowledge within the restricted study timeframe. In fact, this phenomenon of switching majors has been found often in transfer students (Aulck and West, 2017); however, the related issues are seldom discussed. We found those students perceived their workloads to be higher because of their irrelevant academic backgrounds. Based on previous transfer students’ experiences, Caporrimo (2007) indicated that universities are unlikely to provide extra support for students with limited background knowledge. The transfer students in this study suggested that, to address this issue, the university could organize some subjects for them to revise the basic knowledge for the new articulated discipline in order to improve the transition. Similar suggestions to improve the articulation of courses were made by Eggleston and Laanan (2001), whereas Melguizo et al. (2011) believed that both community colleges and universities should provide students with sufficient academic preparation to enhance their adjustment. The implication is that universities may need to revamp subjects regularly in order to maintain smooth transition processes as well as to align with industry expectations.

The transfer students expressed disappointment that their expectations of enriched university life were not realized due to their heavy workloads and the restricted social circle. Transfer students often experience higher workloads (Wang et al., 2010; Mehr and Daltry, 2016; Cheung et al., 2020d), which Walker et al. (2016) attributed to the lack of credit transfer. They found that some credits they had gained in community college could not be transferred to the university, and therefore they had to take the extra credits to fulfill the graduation requirement. This loss of credits during the transition from community college to university is common (Monaghan and Attewell, 2015). Moreover, the subject assessment requirements tend to be more demanding than in their past community college studies (Chan et al., 2021). We also found that non-alignment of the learning content between community college and bachelor’s programs might be another possible reason to increase their workloads. Basically, the heavy workloads for transfer students are also exacerbated by the restricted study timeframe of the “2 + 2” transfer pathway (Ching et al., 2020). Hence, the transfer students interviewed in this study suggested modifying the study duration, for example extending their articulation programs. Yet, extending the duration for transfer students might be a challenge due to the current fixed 2-year government funding source (University Grants Committee (UGC), 2019).

On the other hand, contrary to their expectations, our transfer students found they spent most of their time on study, and thus lacked time for social life (consistent with Townsend and Wilson, 2009). Their lower participation in social activities in university is caused primarily by the heavy workload, which reduces their enthusiasm to participate in social activities, and leads eventually to less social involvement in university (Wang and Wharton, 2010; Ching et al., 2021). The transfer students interviewed in this study also revealed that their restricted social circle was another challenge to their social adjustment and involvement in the new environment. We found that they faced difficulties fitting into the mainstream students’ social circles and felt more comfortable to be with their transfer student counterparts. The challenges with social adjustment faced by transfer students have been reported in previous studies (Britt and Hirt, 1999; Townsend and Wilson, 2009; Reyes, 2011). In other countries, like the US, transfer students are generally older than mainstream students, so this age gap might be a reason why it is difficult for them to enter the younger social circles (Townsend and Wilson, 2006; Fematt et al., 2021). In contrast, there are no significant age differences between these two groups of students in Hong Kong (Cheung et al., 2020c). This implies that the challenges faced by transfer students in social adjustment to university are not only caused by an age difference between themselves and the mainstream students. The transfer students in our study reflected that mainstream students have already established many friendships and social circles during their first 2 years of study in the university. This view was aligned with the finding reported by Townsend and Wilson (2006). It also relates to the finding that it is harder for transfer students to establish social connections on campus through extra- and co-curricular activities than do mainstream students (Massi et al., 2012). On the other hand, the transfer students indicated that they find it easier to connect with their transfer student peers. Similarly, United States transfer students have suggested grouping transfer students together to help them to build up a connection in university (Townsend and Wilson, 2009). The transfer students in this study explained that similar experience and background is the major reason for them to develop friendships with their transfer student peers. It has been shown that transfer students perceive disparities with mainstream students and negative impacts of the stigma they feel as “transfer students” (Laanan, 2000; Caporrimo, 2007; Laanan et al., 2010; Moser, 2013; Wong and Tse, 2017; Cheung et al., 2020c). Thus, it is understandable that transfer students have a preference to be with their transfer student peers. Indeed, social engagement may exhibit via collaborative learning and faculty-student interaction. Transfer students in our study proposed that universities and faculties can take initiatives to improve their social adjustment and enhance their connection with their mainstream student counterparts. This view is consistent with that of Lester et al. (2013), who reported transfer students expecting to establish positive connections with their classmates and looking for peers for teamwork or discussion in both physical and virtual classrooms. In addition, Zilvinskis and Dumford (2018) pointed out that supportive learning environments and teaching pedagogies foster the level of student engagement, whereas Lopez and Jones (2017) indicated that universities should take the responsibility to increase the interactions between transfer students with their peers and faculty in both in and after class as this can benefit to their social adjustment.

Another noteworthy challenge the transfer students experienced was the partial administration arrangement and insufficient support from the university. Our findings showed that several administrative arrangements were not designed for transfer students, like overseas exchange and the subject registration system. We found that participating in the overseas exchange programs seemed to be impossible for the transfer students, and this is consistent with findings from past research studies (Ching et al., 2020, 2021). Yet, the challenge of course registration is not the only problem for transfer students in Hong Kong. It has been reported that some transfer students in the United States also found they were unable to select many subjects because the deadline for selection was before their enrollment (Eggleston and Laanan, 2001), similar to the experience described by our transfer students. In fact, Walker and Okpala (2017) determined that transfer students noticed the disparities between themselves and mainstream students and suggested that university administrators recognize their different needs. Transfer students often feel they are receiving inadequate support from their universities (Eggleston and Laanan, 2001; Kodama, 2002; Cheung et al., 2020c), and want to obtain more resources from their universities (Tobolowsky and Cox, 2012; Walker and Okpala, 2017). As a matter of fact, they have been neglected and labeled as not requiring extra assistance, due to the perception that their previous experience in community college was enough (Beckenstein, 1992), as was the case reported in our interviews. Furthermore, Wang et al. (2010) pointed out that transfer students used fewer support services and had lower awareness of the support services available. These authors explained that different student backgrounds might be a reason for the transfer students having lower awareness levels. They have limited access to information about how to navigate the new institution (Townsend and Wilson, 2006). This was similar to the findings of the current study, in which the students who had transferred from different community colleges felt they were not familiar with the new learning environment and thus had lower awareness of the support services and resources.

### Implications

This manuscript has provided a foundation for future research. The transfer students perceived that the whole transition process was far from meeting their expectations and they had encountered a number of challenges. Recognizing and tackling the in-between challenges of transfer students are essential for them to adopt the new university life (Bhabha, 1996; Dai, 2020; Dai and Hardy, 2021). In order to make a smooth transition and improve transfer students’ retention or “belongingness” to their universities, a “TRANSFER” framework has been designed to suggest possible strategies and recommendations. In this framework, “T” refers to “Technological Support”; “R” refers to Revamping Programs’; “A” refers to “Academic Support”; “N” refers to “Networking”; “S” refers to “Subject registration”; “F” refers to “Facilities”; “E” refers to “Exchange”; and “R” refers to “Resources.” The TRANSFER’ framework mainly focuses on the qualitative analysis to elaborate on perceptions and challenges of transfer students from community college to university in a Chinese educational context. Nevertheless, it gives a theoretical framework for educators and researchers to carry out a large-scale survey. As such, in-depth analysis and comprehensive study of the Asian higher education sector will be achieved in the forthcoming years. This will be helpful in identifying the features of transfer students and implementing appropriate strategies in fostering transfer students to prepare for the future.

#### Transferring Credit

It would be useful to have an integrated online platform listing the subject mapping between community college and university programs, providing information about the subject credit transfer criteria, requirements, and procedure (Cheung et al., 2021). The platform will be an open access system which transfer students or potential transfer students can access without restriction, to enhance the transparency of credit transfer subjects.

#### Revamp the Programs

Close and periodical communication between community college and university program leaders is essential, with the aim to improve subject or program alignment to prevent credit loss, and duplication of learning. The programs in both institutions should be revised based on the students’ feedback, industrial practitioners’ feedback, study load and changes to curricula on both sides.

#### Academic Support

Transfer students have unique needs which mainstream students might not have. An orientation particularly for transfer students during admission can be arranged to prepare them for the challenges ahead and plan how to tackle these challenges with available resources provided. As well, the university may provide more support for their career advancement, such as inviting alumni as peer mentors to share the latest news from the industry as well as their study experiences.

#### Networking

Social networking and interaction is another unique need of transfer students. The program leaders may consider planning common timeslots for transfer and mainstream students to take core subjects together. Moreover, universities may provide more support through various peer activities for transfer and mainstream students to break the repetitive cycle and reduce the labeling effect of transfer students as “inferior.”

#### Subject Registration

Transfer students have to fulfill the graduation requirement by completing the general education requirement subjects within 2 years rather than the four that mainstream students have. As mentioned previously, they have limited subject choices since they are the last group of students in the system. The university may need to allocate some places specially for transfer students in the subject registration system.

#### Facilities

Transfer students only have 2 years to complete their university studies. They have to adapt to the new environment quickly to facilitate their transition. The university may use different effective formal and informal communication channels to inform transfer students about the facilities available, for instance through orientation programs specifically for transfer students, the university website, and social media.

#### Exchange

The university may modify the settings of overseas programs, for example by shortening the exchange duration and increasing the credit transfer agreements with overseas universities, to increase the flexibility for transfer students to join exchange programs.

#### Resources

The university may need to allocate more resources to support transfer students. Universities should provide extra assistants to improve the transfer students’ transition experiences, such as organizing a series of transfer orientation and campus visits, increasing the numbers of places available in hall accommodation for transfer students and establishing a supporting center for them. As well, academic staff should be well informed about transfer students’ unique needs and provide appropriate support.

### Limitations

This manuscript has provided a foundation for future research. There were some limitations to our research. First, our study only concentrated on the “Engineering and Science” discipline. Future research may consider comparing students from other disciplines, for instance “Business and Hospitality,” “Language and Communication,” and “Social Sciences and Design.” This may possibly enable the research findings to be generalized and strengthen our understanding of similarities and differences in transfer processes in different disciplines. Second, self-reported data were adopted that could potentially rely on the participants’ accuracy and inclination to answer. The participants may have been unwilling to report real human behavior due to potential individual re-percussions or a lack of knowledge. Third, due to the participants’ availability, two sessions of individual interviews were conducted, which might have reduced the dynamics of the discussion and elicitation of ideas. Last, the main data were collected from students. Further research may gather data from various other stakeholders, including policymakers, service providers, government bodies, and educators, via focus group interviews to generate comprehensive data and achieve broader viewpoints for further analysis.

## Data Availability Statement

The original contributions presented in the study are included in the article/supplementary material, further inquiries can be directed to the corresponding author.

## Ethics Statement

The studies involving human participants were reviewed and approved by the Human Subjects Ethics Sub-Committee at the Hong Kong Polytechnic University (HSEARS20170808003). The patients/participants provided their written informed consent to participate in this study.

## Author Contributions

Y-yL and KC: conceptualization and validation. Y-yL: methodology. Y-yL and CH: formal analysis and writing-original draft preparation. Y-yL, YT, NY, and KC: data curation. YT, NY, WK, CH, and KC: writing-review and editing, and project administration. KC: funding acquisition. All authors have read and agreed to the published version of the manuscript.

## Conflict of Interest

The authors declare that the research was conducted in the absence of any commercial or financial relationships that could be construed as a potential conflict of interest.

## Publisher’s Note

All claims expressed in this article are solely those of the authors and do not necessarily represent those of their affiliated organizations, or those of the publisher, the editors and the reviewers. Any product that may be evaluated in this article, or claim that may be made by its manufacturer, is not guaranteed or endorsed by the publisher.
